# Effects of chamber shapes on maneuverability and control property of endoscope-support soft actuators

**DOI:** 10.3389/fbioe.2023.1319922

**Published:** 2023-12-14

**Authors:** Yuxi Lu, Zhongchao Zhou, Pablo Enrique Tortos Vinocour, Shota Kokubu, Tatsuo Igarashi, Wenwei Yu

**Affiliations:** ^1^ Department of Medical Engineering, Graduate School of Engineering, Chiba University, Chiba, Japan; ^2^ Center for Frontier Medical Engineering, Chiba University, Chiba, Japan

**Keywords:** medical robotics, endoscope-support soft actuator, pneumatic chamber, frictional interaction, finite element analysis, maneuverability, control property

## Abstract

**Introduction:** Minimally Invasive Surgery (MIS) offers targeted surgical access with reduced invasiveness; however, the maneuverability challenges of traditional instruments in this domain underscore the need for innovative solutions. Soft actuators activated by fluids or gases present a promising strategy for augmenting endoscopic capabilities, thereby enhancing the surgical precision in MIS. This study aimed to explore the intricate dynamics of the interactions between soft actuators and endoscopes, with an emphasis on the pivotal role of cross-sectional chamber shapes. While previous studies have touched on the influence of chamber shapes on bending properties, we provide a comprehensive exploration. We explore how these shapes modulate friction forces, which in turn influence the interactions governing bending, response, and stiffness adjustability, all of which are essential for enhancing endoscope maneuverability in MIS contexts.

**Methods:** A novel bilateral symmetrical air chamber design was adopted to investigate various chamber shapes. We employed finite element analysis (FEA) simulations followed by prototype testing to evaluate the interactions driven by these chamber shapes and to discern their impact on actuator properties. Recognizing the pivotal role of friction in these interactions, we conducted dedicated friction experiments. These experiments further deepened our understanding of the relationship between chamber shape and friction, and how this synergy influences the properties of the actuator.

**Results:** Our findings showed that actuators with wider chambers generate larger friction forces, thereby enhancing the interaction and improving the bending, response, and stiffness adjustability. Additionally, the soft actuator significantly improved the maneuverability and bending radius of the endoscope, demonstrating enhanced navigation capabilities in complex environments.

**Discussion:** The shape of a cross-sectional chamber plays a pivotal role in designing soft actuators for MIS applications. Our research emphasizes the importance of this design component, offering key insights for the development of endoscope-supporting soft actuators that can effectively handle intricate actuator-endoscope interactions, thereby enhancing surgical outcomes.

## 1 Introduction

Minimally Invasive Surgery (MIS) allows surgical procedures through minimal incisions or natural orifices to access target sites with minimal invasiveness. However, the constrained and unstructured workspace within MIS procedures poses challenges to the maneuverability of traditional rigid instruments, thereby limiting access to all regions of the surgical site (Anderson et al., 2016). These instruments, often driven by multiple fiber groups, encounter substantial interference among these fibers, which in turn limits their motion range and hampers their maneuverability (Zhu et al., 2022). Additionally, the inherent stiffness of rigid instruments, which cannot be adjusted, poses risks of causing damage or exerting excessive pressure on surrounding tissues in unstructured environments (Simaan et al., 2018). This limitation in maneuverability has frequently been highlighted by clinicians. Additionally, the development of small, multifunctional, rigid surgical instruments is challenged by current manufacturing techniques, both in terms of feasibility and cost (Rodr et al., 2006). In the realm of control engineering, “maneuverability” encompasses three different concepts: the control degree of freedom (DOF) of a system (Craig, 2006), the ability to induce an increment in steady motion (Yokokohji and Yoshikawa, 1990) in response to control variations (akin to changing its pose), and the extent to which the response of an actual system matches the ideal state; for example, the state of a master device in a master-slave operation system (Cook, 2013). For the scope of our study, we adopted the first interpretation (Craig, 2006), focusing specifically on the control DOF as it aligns closely with our research objectives.

In response to the limitations of maneuverability in traditional rigid instruments, soft robotics, which is characterized by the use of highly compliant materials, has emerged as a promising avenue to enhance maneuverability in healthcare applications (Maghooa et al., 2015; Maghooa et al., 2015; Decroly et al., 2020). These robots, particularly those adept at unstructured environments and human interactions, can deform around their surroundings, minimizing potential damage to adjacent tissues (Laschi and Cianchetti, 2014). Among the various soft robotics designs, soft actuators constructed from elastomeric materials and activated by pressurized fluids or gases have gained significant attention. They are designed for specific motions, such as translation or rotation through axial or radial deformation (Connolly et al., 2015; Wang et al., 2017; Polygerinos et al., 2015; Wang et al., 2021) However, the application of these soft actuators in MIS, especially as support for endoscopes, faces a set of challenges.

In MIS, one approach for utilizing soft actuators is to integrate them directly into the system alongside soft surgical instruments, notably endoscopes, to leverage their compactness, low hardness, and bidirectional bending capabilities (Runciman et al., 2019). This integration aims to mitigate the effects of the stiffness of internal instruments during actuation and ensure stable operability by preventing relative sliding. However, these designs have limitations and are yet to be implemented in real surgeries. They cannot accommodate conventional endoscopes, are expensive, and lack adaptability for various surgical scenarios (Runciman et al., 2019).

Another approach to soft actuators in the realm of MIS involves using them as bidirectional bending access channels for MIS instruments (Low et al., 2015; Abidi et al., 2018). These actuators have broad internal access channels designed to accommodate both surgical instruments and endoscopes. This design feature is essential for facilitating the interactions between soft actuators and endoscopes. By enhancing DOF and/or the range of motion, soft actuators enable the MIS to access hard-to-reach areas within the body, thereby ensuring safe navigation between organs.

Notably, several companies, including Ambu Inc. and Olympus Corp., have introduced flexible soft endoscopes. These instruments, equipped with a single DOF for lens rotation within the human body, are equipped with an access channel for surgical tools, and have found their niche in surgeries, such as thoracoabdominal and bladder-related procedures. However, their limited DOF often hampers their maneuverability, especially in intricate procedures, such as transcystic resection of the prostate (Chang et al., 2022), where the endoscope needs to navigate through the bladder, adjust its overall position, maintain its posture, and perform precise surgical operations. The complexity and precision required for such procedures highlight the need for multiple DOF. Additionally, these flexible endoscopes lack the ability to adjust their stiffness in response to varying surgical conditions, limiting their stability and increasing the operational challenge for surgeons. Moreover, any attempt to enhance their DOF involves complex and costly modifications to their mechanical and control systems, impacting their reliability and usability. Soft actuators, specifically those used as bidirectional bending access channels for MIS instruments (Abidi et al., 2018), can complement these instruments and enhance their DOF and the range of motion for a broader array of surgeries.

The determination of the material properties of soft actuators, specifically those used as bidirectional bending access channels for MIS instruments, is challenging. Many of these actuators are constructed from elastomeric materials, such as silicone rubbers, which typically possess a shore hardness in the range of 10–30 A (Xavier et al., 2021). In contrast, mainstream endoscopes from certain companies, such as Ambu Inc. and Olympus Corp., are made of relatively stiffer materials for various functionalities, including damage prevention, water resistance, and signal transmission, resulting in an overall stiffness of approximately 50 A. This stark contrast in material properties considerably surpasses that of soft actuators and can pose challenges during their interaction, with factors, such as the contact surface area, inherent stiffness of the endoscope, and friction, being vital determinants of this dynamic. The significant role of friction not only affects the bending and response properties of the actuator (Runciman et al., 2019), but also leads to relative sliding between components owing to a disparity in stiffness. In addition to causing actuation delays, such interactions can further influence other essential actuator properties.

This interaction highlights another crucial aspect: the necessity for soft actuators to adjust stiffness, ensuring a stable surgical view of the dynamic complexities of surgical environments. To address this requirement, a bilateral symmetrical air chamber design has been recognized, as supported by research (Stilli et al., 2014; Lu et al., 2023), as an effective solution. This design ensures real-time adaptability and is notable for its inherent efficiency and practicality. It is important to emphasize that the interactions between the endoscope and soft actuator, stemming from the differences in material properties, might further influence the stiffness adjustability, an aspect that merits further exploration.

To continue our exploration of the intricate design of soft actuators, we must consider the specific constraints imposed by the environment and equipment in the MIS. The size and materials used in MIS instruments are inherently limited. These constraints, especially those concerning the outer and inner diameters and materials of the actuator, underscore the significance of another key component: the cross-sectional chamber. Among the various influential variables, shape emerges as a crucial design component that plays a significant role in modulating the interaction between the actuator and the endoscope, thereby influencing the overall performance of the actuator-endoscope system. Previous studies (Suzumori et al., 2007; Elsayed et al., 2014; Low et al., 2015; Abidi et al., 2018; Deimel and Brock, 2016) have highlighted the effects of various chamber shapes, such as circular (Low et al., 2015; Abidi et al., 2018), rectangular (Abidi et al., 2018), semicircular (Suzumori et al., 2007; Wang et al., 2021), and fake crescent (Suzumori et al., 2007; Wakimoto et al., 2009), on the bending properties of soft actuators. However, the broader implications of enhancing the navigational process of endoscopes during MIS (Abidi et al., 2018) remains unknown.

A notable gap exists in our holistic understanding of the broad implications of these chamber shapes. The prevailing literature, while extensively exploring bending properties, has largely overlooked the significance of chamber shapes in the interaction dynamics between the endoscope and the soft actuator (Suzumori et al., 2007; Elsayed et al., 2014; Runciman et al., 2019). Given the integral role these interactions play in determining the bending properties, response properties, and stiffness adjustability—the latter being optimized by a bilateral symmetrical air chamber design—it becomes imperative to investigate the influence of chamber shapes on such interactions.

Moreover, the influence of the chamber shape, especially during endoscope insertion, on the response properties and stiffness adjustability remains underexplored. Addressing this knowledge gap is compelling and opens an avenue to amplify the benefits offered by different innovations, such as the bilateral symmetrical air chamber design. A more comprehensive exploration of chamber shapes could elucidate their impact on both response properties and stiffness adjustability with the ultimate goal of enhancing the maneuverability and control of endoscopic instruments in MIS scenarios.

Central to our study is the interaction between the soft actuator and endoscope, a dynamic that is fundamental for enhancing the efficiency and safety of MIS procedures. Our study aimed to understand how the design of these cross-sectional pneumatic chambers can affect critical attributes, such as bending properties, response properties, and stiffness adjustability, which in turn influence the maneuverability (Craig, 2006) and control of endoscope-supporting soft actuators. Specifically, we focused on optimizing the control of multi-DOF of the endoscope by designing a soft actuator. We prioritized refining control over the existing DOF to ensure a more effective range of motion. This nuanced approach seeks to elevate the functionality of both individual soft actuators and the composite endoscope-soft actuator system. Delving deeper into the mechanisms of interaction, we envision significant potential not only in broadening the design parameters but also in enhancing the response and stiffness adaptability of soft actuators. This approach distinguishes our research from conventional studies, offering a unique perspective. To further underscore this distinctiveness and provide a clear understanding of how our research advances the field, we have added a comparison table (Table 1) juxtaposing the key aspects of this research with previous related studies.

**TABLE 1:** Proposed study vs. previous studies.

Features	Proposed study	Previous studies	References
Focus	Interaction dynamics between pneumatic chamber designs and endoscope-soft actuator systems, with an emphasis on friction as a pivotal factor	Primarily focused on bending characteristics and basic functionality of soft actuators	Runciman et al. (2019); Elsayed et al. (2014); Abidi et al. (2018)
Design Innovation	Bilateral symmetrical air chamber design with hollow spaces, optimized for integrating with clinical endoscopes and enhancing maneuverability	Traditional designs emphasizing limited functionality, lacking integrated space for endoscopes	Stilli et al. (2014)
Chamber Shapes	Extensive exploration of seven diverse chamber shapes to determine their impact on actuator performance and identify optimal designs for specific surgical needs	Focus on limited chamber shapes, neglecting the potential of diverse designs to enhance actuator functionality and surgical efficiency	Suzumori et al. (2007); Elsayed et al. (2014)
Objective	Enhancing bending property, response property, and stiffness adjustability in soft actuators for improved the maneuverability and control property of endoscope	Focus on bending and physical properties of soft actuators	Elsayed et al. (2014); Abidi et al. (2018)

To elucidate our approach, we modeled endoscope-supporting soft actuators with a simplified structure and single DOF. A notable feature of our design is its innovative antagonistic structure that confers enhanced stiffness adjustability to soft actuators. In particular, our design features bilateral symmetrical air chambers separated by hollow spaces. This hollow space is not only a structural novelty; it also serves a functional purpose by providing an insertion channel for endoscopes and other surgical instruments. In contrast to conventional antagonistic structures used in soft actuator applications (Stilli et al., 2014), where chambers are typically positioned adjacently without any intervening space, our configuration offers minimized interference between the chambers owing to spatial separation.

For a comprehensive study, finite element analysis (FEA) simulations were employed to incorporate seven distinct pneumatic chamber shapes into the model: circular, semicircular, square, rectangular, fake crescent, long fake crescent, and crescent. FEA is a valuable tool that is widely used in soft actuator research (Wakimoto et al., 2009; Rus and Tolley, 2015; Marchese et al., 2015; Heung et al., 2020; Lee et al., 2017; Agarwal et al., 2016). Owing to its inherent advantages, including cost-effectiveness, design flexibility, and predictive accuracy (Connolly et al., 2015; Cao et al., 2018; Duriez, 2013; Marechal et al., 2021), FEA provides in-depth insights into the internal structural dynamics of soft actuators during bending. It can dissect the effects of various parameters, including chamber shape, material properties, and boundary conditions, thus guiding the optimized design of soft actuators tailored for medical robotics applications.

Through meticulous finite element analysis (FEA) and subsequent prototype testing, we investigated the interactions induced by different chamber shapes in soft actuators. Understanding these interactions and their influence on the relevant properties, such as bending, response, and stiffness adjustability, is essential for advancing the design of soft actuators for endoscopic applications.

Furthermore, our study revealed that friction is a pivotal factor in these interactions. We observed that the actuators with wider chamber widths generated larger frictional forces, thereby enhancing the interactions between the components. This increase in friction not only improved the bending properties of the soft actuator but also optimized its response properties and stiffness adjustability. These findings underscore the criticality of the chamber shape and friction in design, proposing avenues for advancements in the design and performance of soft actuators for MIS applications.

Finally, the evaluation of bladder models confirmed the practical advantages of the soft actuator, highlighting its potential for enhancing the operational range and bending properties of the endoscope.

## 2 Materials and methods

### 2.1 Hypothesis

Our primary hypothesis posited that the friction forces at the interface between the actuator and endoscope are vital in determining the performance of the actuator. While reduced friction is intuitively expected to enhance the performance owing to less resistance from the endoscope, heightened friction might also have potential benefits. Furthermore, the control performance of the actuator is closely related to its bending properties, both of which are influenced by the shape-induced deformation patterns of the chamber and the internal stress distribution.

Based on this hypothesis, our experimental methodology and subsequent analyses were designed to elucidate the intricate relationships between the design nuances and performance metrics in soft actuators.

### 2.2 Design for soft actuators

**FIGURE 1:**
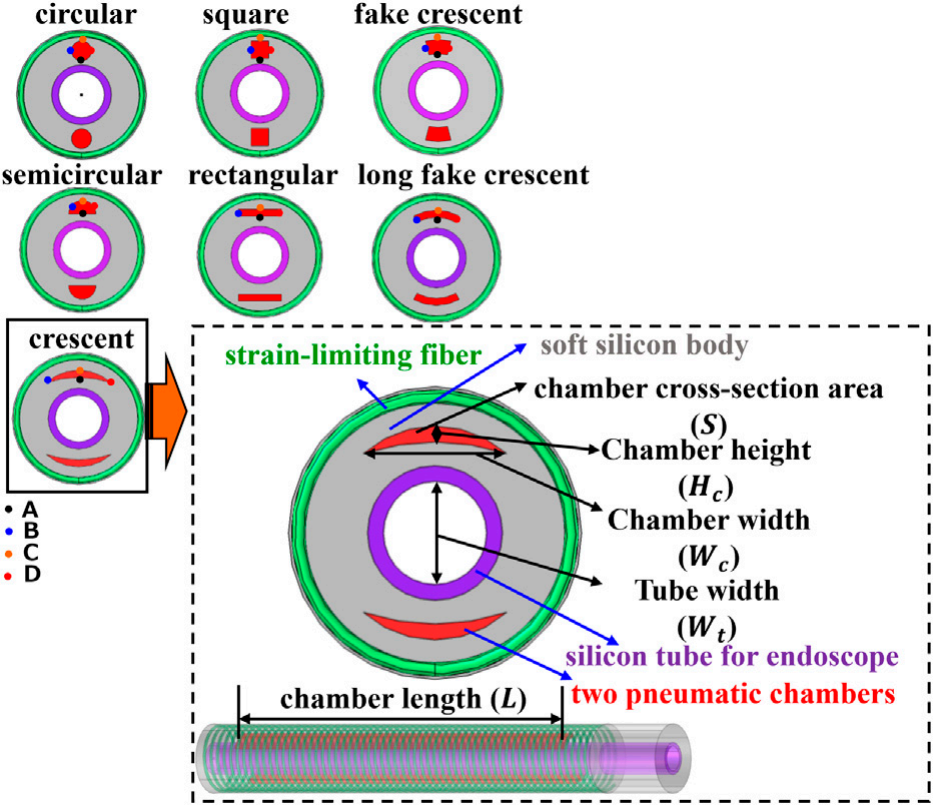
Investigated pneumatic chamber designs and parameters.

To advance our understanding of the role of chamber shapes in soft actuator performance, we expanded our investigations to encompass a wider range of chamber shapes, compared with that in previous studies (Suzumori et al., 2007; Elsayed et al., 2014). Figure 1 shows the shapes of the investigated pneumatic chambers: circular, square, fake crescent, semicircular, rectangular, long fake crescent, and crescent. The parameters of each pneumatic chamber are listed in Supplementary Table S1. For consistency in our analyses, we maintained uniform cross-sectional areas and lengths across all the pneumatic chambers. Furthermore, the central point of each chamber’s cross section was aligned with the midpoint of the outer wall of the soft actuator and endoscope tube. The soft actuator was composed of two symmetrical pneumatic chambers, a strain-limiting fiber, a silicon tube for the endoscope (radius: 4.25 mm, length:140 mm, thickness:1 mm), and a soft silicon body (radius: 8.25 mm, length:120 mm). Finally, the root part of the actuator (radius: 8.25 mm, length:20 mm) was fixed. The silicon tube allowed the endoscope to be carried and could be used as an end effector on a steerable catheter to reach locations inaccessible to the endoscope, thus increasing the surgeon’s dexterity. A strain-limiting fiber (major radius: 8.65 mm, minor radius: 0.4 mm, axial pitch:2 mm, number of turns:59) with helix restraint was externally added.

### 2.3 FEA modelling

A quasi-static numerical finite element model was developed using COMSOL Multiphysics 6.0 ^®^ to investigate the effect of different cross-sectional shapes of pneumatic chambers on the bending and response properties of soft actuators. The detailed modeling process is as follows:

• Modeling Software and Interface: The Solid Mechanics Interface of the Structural Mechanics Module of COMSOL Multiphysics 6.0^®^ was used to model the soft actuators.
• Simulation Conditions: Boundary Load conditions were used to simulate the input air pressure. These conditions were selected to accurately represent the physical application of pressure within the pneumatic chambers during actual usage.
• Geometry and Parameters: The geometry of the numerical model is shown in Figure 1. The parameters of the soft actuator (Supplementary Table S2) were based on the designed geometry, with the varition being the cross-sectional shape of the pneumatic chamber between different samples.
• Boundary Constraints: A fixed boundary constraint was applied to the terminal cylinder surface (radius: 8.5 mm, length: 20 mm) of the silicon body.
• Material Modeling: A third-order incompressible Yeoh model was used to model the hyperplastic silicon body. This model choice was informed by its accessibility as a constitutive model in the FEA software and its proven performance under high strains. The material parameters were determined using a nonlinear least-squares optimization method with the Levenberg–Marquardt algorithm by fitting the experimental data.
• Model Specifications: The strain energy density function ( $W_{siso}$ ) of the Yeoh Model is presented as (1).

$$W_{siso}=c_{1}\left(\bar{I_{1}}-3\right)+c_{2}{\left(\bar{I_{1}}-3\right)}^{2}+c_{3}{\left(\bar{I_{1}}-3\right)}^{3}$$ (1)

where *I*_1_ denotes the strain invariant. The Soft Robotics Materials Database^33^ characterization of Dragonskin 10 MEDIUM and Dragonskin 30 was implemented with the coefficients *c*_1_ = 0.0547 *MPa*, *c*_2_ = 0.0002 *MPa*, *c*_3_ = 0 *MPa* and *c*_1_ = 0.1536 *MPa*, *c*_2_ = 0.0016 *MPa*, *c*_3_ = 0 *MPa*. The densities of DragonSkin 10 MEDIUM and DragonSkin 30 were the same at 1070 $\text{kg}/m^{3}$ .

• Actuator and Fiber Modeling: In the case of the actuator, the strain-limiting fiber (major radius: 8.65 mm, minor radius: 0.483 mm, axial pitch: 2 mm, number of turns: 59) with helix restraint was externally added. It was modeled by a liner elastic material, having a Young’s modulus of E=31067 MPa , a Poisson’s ratio of υ=0.36 , and a density of 1,440 $\text{kg}/m^{3}$ . This fiber addition is critical for limiting strain and emulating the real-world operational conditions of the actuator.
• Meshing Strategy: For the mesh, all the components of the actuator were modeled using solid tetrahedral quadratic hybrid elements. For the thin-fiber windings, a combination of tetrahedral quadratic hybrid elements and quadratic beam elements were utilized to ensure precise representation of the physical characteristics.

We have included the COMSOL simulation files as a Supplementary Material for further reference and validation.

### 2.4 Measurement in FEA simulation

**FIGURE 2:**
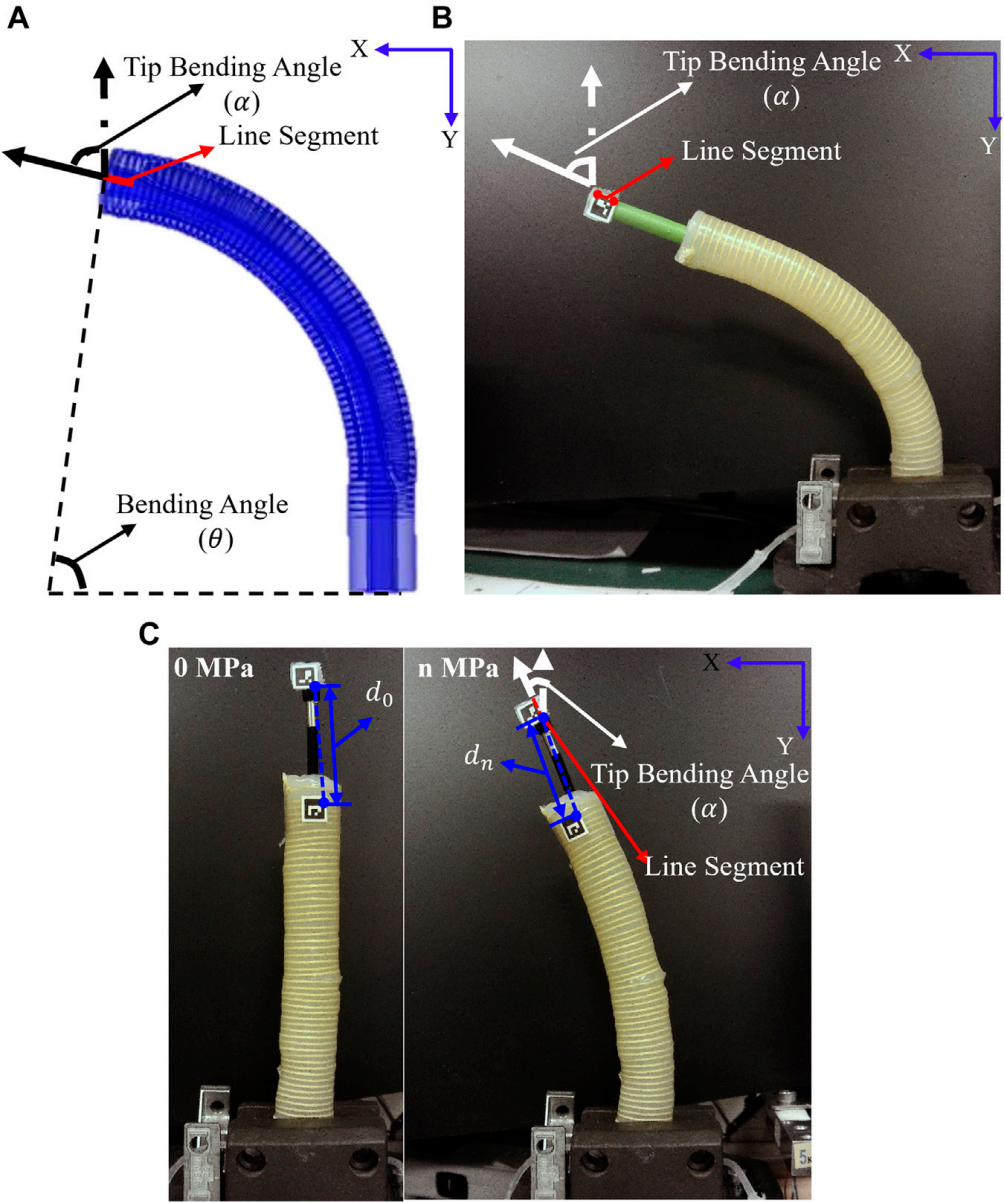
Assessment of soft actuator **(A)** with FEA simulations **(B)** for free bending **(C)** and for bending with inserted endoscope.

A series of FEA simulations were performed to investigate the bending behavior of all soft actuators with different cross-sectional shapes of the pneumatic chambers using the COMSOL Multiphysics software. Simulations were conducted for each soft actuator with only one pneumatic chamber, and the pressure loads were varied in the range of 0–200 kPa. The measurement method for the soft actuator in the FEA simulations is shown in Figure 2A.

The tip bending angle ( α ) of all soft actuators was determined from the results of the deformed shape at each pressure. To analyze the influence of different cross-sectional shapes of pneumatic chambers on the tip bending angle ( α ), the change ratios of chamber length $R_{L}$ , chamber width ( $R_{w}$ ), chamber height ( $R_{H}$ ), and chamber cross-sectional area ( $S_{c}$ ) were measured at all pressures. These ratios were defined by Eq. 2.

$$\begin{array}{c} R_{L}\left(n\right)=\frac{L\left(n\right)-L\left(0\right)}{L\left(0\right)}\times 100\%,R_{w}\left(n\right)=\frac{w_{c}\left(n\right)-w_{c}\left(0\right)}{w_{c}\left(n\right)}\times 100\%,\\ R_{H}\left(n\right)=\frac{H_{c}\left(n\right)-H_{c}\left(0\right)}{H_{c}\left(n\right)}\times 100\%,R_{C}\left(n\right)=\frac{s_{c}\left(n\right)-s_{c}\left(0\right)}{s_{c}\left(n\right)}\times 100\% \end{array}$$ (2)

L is the chamber length, $w_{c}$ is the chamber width, $H_{c}$ is the chamber height, S : the cross-sectional area of the chamber, and n is the air pressure.

Tube widths ( $W_{t}$ ) were measured at various pressures. At the same time, we also measured the stress distribution of the cross-sectional of each soft actuator.

A previous study reported that the bending angle is proportional to the increased volume of the chamber ( ∆V ), as shown by (3) (Agarwal et al., 2016).

α∝ΔV (3)

### 2.5 Measurement of free bending in prototype experiments

To verify the results of the FEA simulations, a series of prototype experiments were conducted to test the bending of soft actuators (circular, rectangular, long fake crescent, and crescent) with different cross-sectional shapes of pneumatic chambers under different pressure conditions of one pneumatic chamber. The fabrication of the soft actuator is described in the “Fabrication of the soft actuator” section in the Supplementary Material. The measurement method used in the experiments is illustrated in Figure 2B. To measure the tip bending angle of the soft actuators accurately, a cylinder (radius: 3.25 mm, length:45 mm) was placed on top of each soft actuator. The soft actuators were held vertically, and an ArUco marker (squared binary marker) was placed at the top of the cylinder. During the bending process, the marker was photographed using a camera (USB8MP02G-SFV, ELP camera, Ailipu Technology Co., Ltd., CN). OpenCV with Python 3.8.12 was used for two-dimensional marker detection and calculation of the tip bending angle of the soft actuators. The tip bending angle was defined by the two points on the right-hand side. To ensure the safety of all the soft actuators, the maximum input air pressure was set to 200 kPa.

### 2.6 Measurement of bending property, control property, and stiffness adjustability of the soft actuator in prototype experiments

The bending property and relative sliding between the soft actuators (circular, rectangular, long fake crescent, and crescent) and the endoscope (radius:3.9/2 mm, hardness:80A, Sanko, JP) were assessed through prototype experiments. To evaluate the effects of the relative sliding and mutual interference between the soft actuator and endoscope during insertion, a measurement method was developed, as shown in Figure 2C. Two ArUco markers (squared binary markers) were placed at the top of the soft actuator and endoscope, and the apparatus was held vertically. The measurement procedure was identical to that used to measure free bending in the prototype experiments. The sliding distance ( $d_{s}$ ) is defined by (4).

$$d_{s}=d_{n}-d_{0}$$ (4)

where $d_{n}$ is the distance between the tip of the soft actuator and the tip of the endoscope at n kPa, $d_{0}$ is the distance between the tip of the soft actuator and the tip of the endoscope at 0 kPa.

Building upon the hypothesis of this study, which emphasized the role of friction in actuator performance, we conducted an experiment to elucidate the effects of friction. A lubricant (CaineZero^®^ Jelly, Fujifilm Corp, JP) was applied to reduce the friction between the outer wall of the endoscope and the inner wall of the tube. The bending properties under high-friction (without lubricant) and low-friction (with lubricant) conditions were compared. The experiments were conducted in the same manner as described for the measurement of the bending property, control performance, and stiffness adjustability of the soft actuator in the prototype experiments.

In alignment with the hypothesis that postulates the influence of the chamber shape on the control performance of an actuator, we performed a series of evaluations. Typical Proportional Integral Differential (PID) controllers were employed to assess the control performance of soft actuators with air chambers of different cross-sectional shapes (circular, rectangular, long fake crescent, and crescent). The control gains were selected through trial and error, aiming for a response with no overshoot and quick recovery. For the circular, fake crescent, long fake crescent, and crescent soft actuators, the control gains were *p* = 0.220, I = 0.130, and D = 0.100; *p* = 0.200, I = 0.105, and D = 0.100; *p* = 0.150, I = 140, and D = 0.100; and *p* = 0.100, I = 107, and D = 0.1, respectively. The desired angle was set at 30° with a maximum input air pressure of 200 kPa for all the soft actuators. An Arduino UNO (Arduino Uno Rev3, ARDUINO CC., United States) integrated circuit served as the primary control chip, with an air pressure regulator SMC ITV0030-2 ML (SMC Pneumatics Ltd., United Kingdom). The air pressure ranged from 0 kPa to 200 kPa. The angular position and velocity data were recorded at a rate of 100 Hz using an Adafruit BNO055 nine-axis sensor. The discrepancy between the actual and desired angles was fed into the designed PID controller to generate the required air pressure. Finally, the maximum bending angle corresponding to identical PID parameters for soft actuators was examined.

To validate the stiffness adjustability, three desired angles of the soft actuators (0°, 45°, and 90°) were set, and varying weights (0.2, 0.4, 0.6, 0.8, 1 N, Tokyo Garasu Kikai Co., Ltd., JP) were placed atop the soft actuators (circular, rectangular, long fake crescent, and crescent) to serve as loads. When the desired angle was 0°, an equal air pressure was applied on both sides of the chamber to increase the actuator stiffness. In conditions where the desired angles were 45° and 90°, the opposite chamber was subjected to an air pressure of 30 kPa, whereas the chamber intended for bending was subjected to an air pressure of (0–200 kPa) that could track the specific desired angle. Consistent with the frictional conditions set (high and low friction) during our experiments on the bending property, we further investigated the effects of high and low friction on the stiffness adjustability when the desired angle was set at 0°. This investigation was conducted with the expectation that an understanding of the nuanced role of friction may influence the stiffness adjustability of soft actuators.

### 2.7 Test for the soft actuator in the bladder model with the flexible endoscope

**FIGURE 3:**
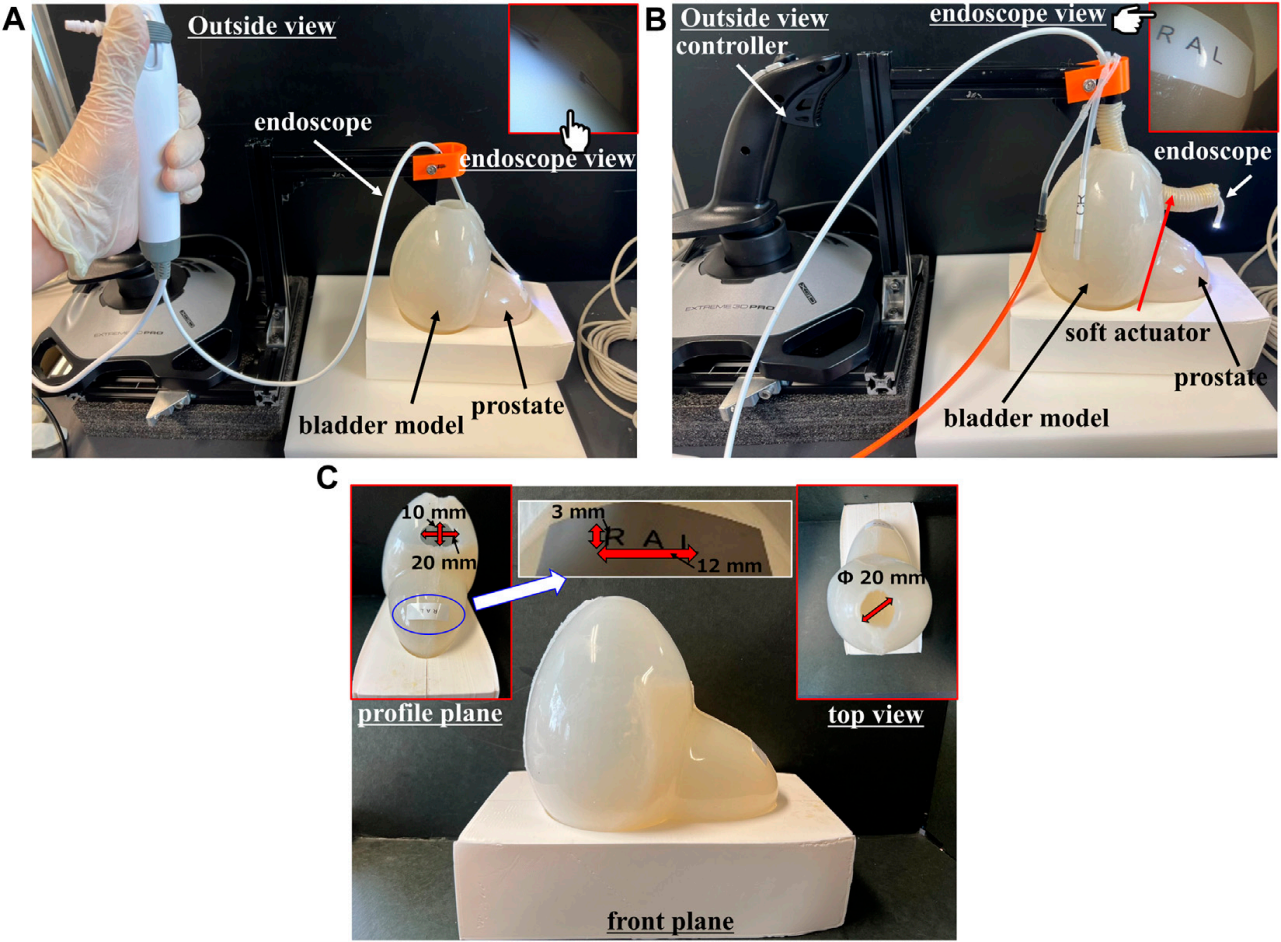
**(A)** Image of testing for flexible endoscope without soft actuator in bladder model **(B)** Image of testing for soft actuator when inserting flexible endoscope in bladder model **(C)** Image of bladder model.

The practicality and operability of the soft actuators were tested in a bladder model by inserting a flexible endoscope (Ambu^®^ aScopeTM 4 Broncho Slim 3.8/a1.2, Ambu Inc., DK) (Figure 3A). The bending of the soft actuators (circular, fake crescent, long fake crescent, and crescent) was controlled using a controller (Logitec Extreme3D Pro, Logicool Co. Ltd., JP). For comparison, tests were conducted using only a flexible endoscope without soft actuators (Figure 3B). The bladder model was fabricated using Dragonskin 10 MEDIUM (Smooth-On, Inc., US), and a square paper was placed in the upper part of the prostate model to test the largest surgical area of all soft actuators. To further test the practicality and operability of the soft actuator with a crescent cross-sectional of the pneumatic chamber, a target surgical area, “L, A, R” was set on the bladder model (Figure 3C). Three experimental persons with no experience in developing soft robots and controllers were set up to confirm the practicality and operability of the soft actuator and controller during the experiments.

## 3 Results

### 3.1 FEA simulations

**FIGURE 4:**
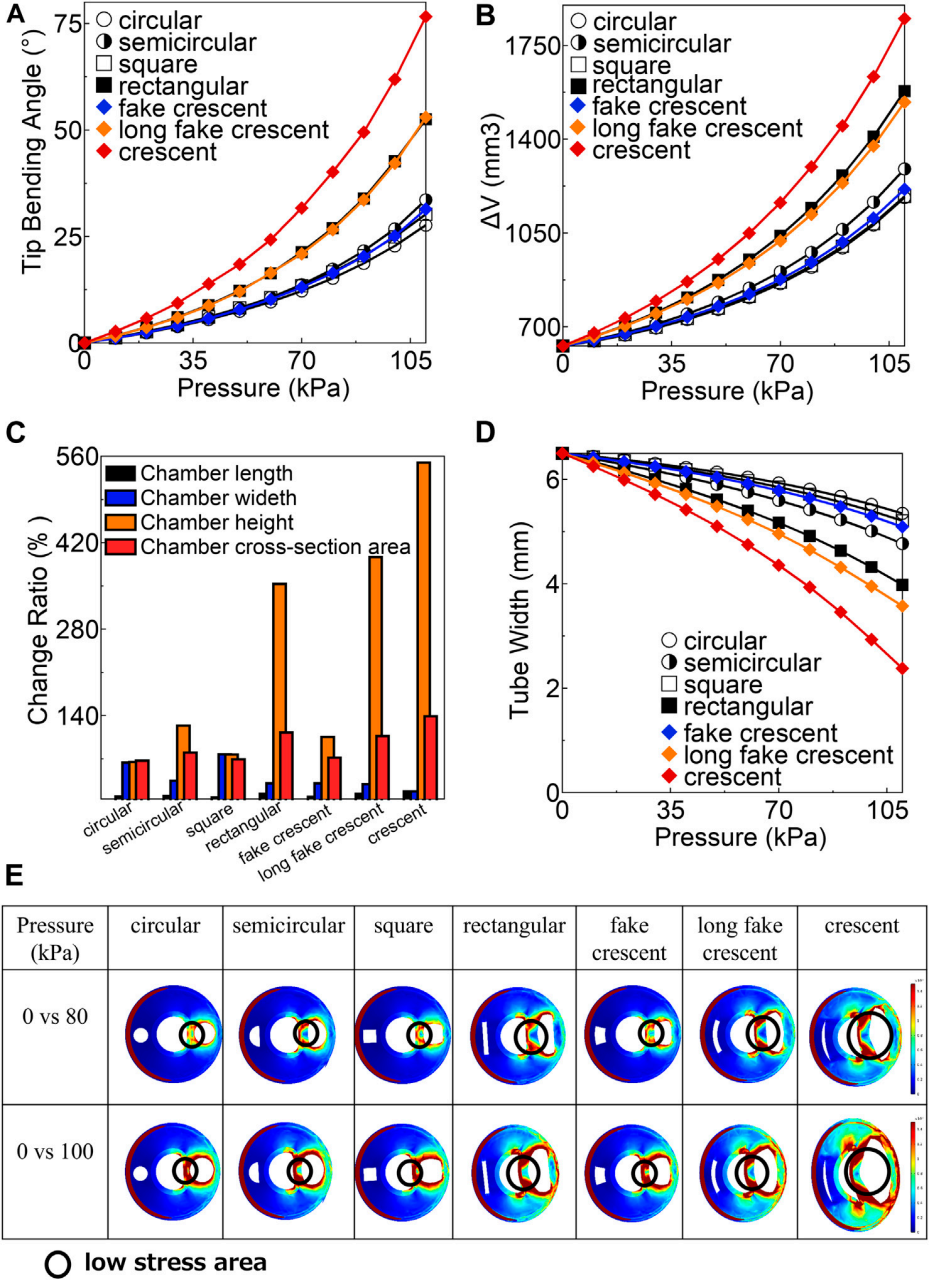
**(A)** The variation of tip bending angle under 110 kPa **(B)** The variation of ∆V under 110 kPa **(C)** Change ratio at 110 kPa **(D)** The variation of tube width under 110 kPa **(E)** The stress distribution of cross-section of all soft actuator at 80 and 110 kPa.

This study presented the variation in the tip bending angle under different air pressures with different cross-sectional shapes of pneumatic chambers, including circular, semicircular, square, rectangular, fake crescent, long fake crescent, and crescent. As depicted in Figure 4A, the soft actuator with a crescent cross-sectional of a pneumatic chamber showed the largest tip bending angle, with the following order of bending angles from largest to smallest: crescent >> rectangular > long fake crescent >> semicircular > fake crescent > square > circular. These findings suggest that the cross-sectional shape plays a pivotal role in influencing bending performance.

The pressure variation of the chamber volume’s change ( ∆V ) with the seven types of pneumatic chamber cross-sectional shapes under 110 kPa is shown and compared in Figure 4B. The tip bending angle of the soft actuator was proportional to the increase in chamber volume ( ∆V ). The change ratios of the chamber length ( $R_{L}$ ), width ( $R_{w}$ ), height ( $R_{H}$ ), and cross-sectional area ( $S_{c}$ ) at a pressure of 110 kPa are shown in Figure 4C. Notably, the change in the chamber height was considerably more pronounced than that in the chamber width.

The hollow structure used for inserting the endoscope caused a significant expansion of the pneumatic chamber on the hollow side. The tube widths in the middle of the chamber for each soft actuator at air pressures ranging from 0 to 110 kPa are shown in Figure 4D. The crescent-shaped chamber exhibited the largest change in tube width.

The stress distribution across the cross-section of all soft actuators at 80 kPa soft and 110 kPa was measured and is presented in Figure 4E. The results revealed that all the actuators had a low-stress area at the bottom of the chamber. Specifically, the crescent-shaped chamber exhibited the most expansive low-stress area. To analyze the effect of low stress on the change of tip bending angle ( ∆θ ), the stress at A, B, C, and D (as referenced in Figure 1) of the inflated chamber at different tip bending angles was measured and presented in Supplementary Figure S1. The results indicated that a wider chamber width (rectangular, long fake crescent, and crescent) led to lower stress at A, whereas a circular chamber resulted in larger stress at A, B, C, and D, compared with the other soft actuators at the same tip bending angle.

### 3.2 Prototype experiments for free bending

Four soft actuators with pneumatic chambers of different cross-sectional shapes (circular, fake crescent, long fake crescent, and crescent) were selected for the prototype experiments based on the tip bending angle results from the FEA simulations. A comparison of the tip bending angle variation at 110 kPa between the simulation and prototype experiments is shown in Supplementary Figure S2. The crescent-shaped chamber exhibited the largest tip-bending angle (crescent > long fake crescent > fake crescent > circular). The bending performance of the soft actuators in the prototype experiments was consistent with that in the FEA simulations, thereby confirming the effectiveness of the FEA simulations. Nevertheless, the prototype experimental results indicated a greater bending behavior than the suggested simulations. This deviation is believed to arise from the material properties in the Soft Robotics Materials Database (Marechal et al., 2021) for the Yeoh model, which align with observations from a previous study (Marechal et al., 2021).

### 3.3 Prototype experiments for bending property, control property, and stiffness adjustability of the soft actuator

**FIGURE 5:**
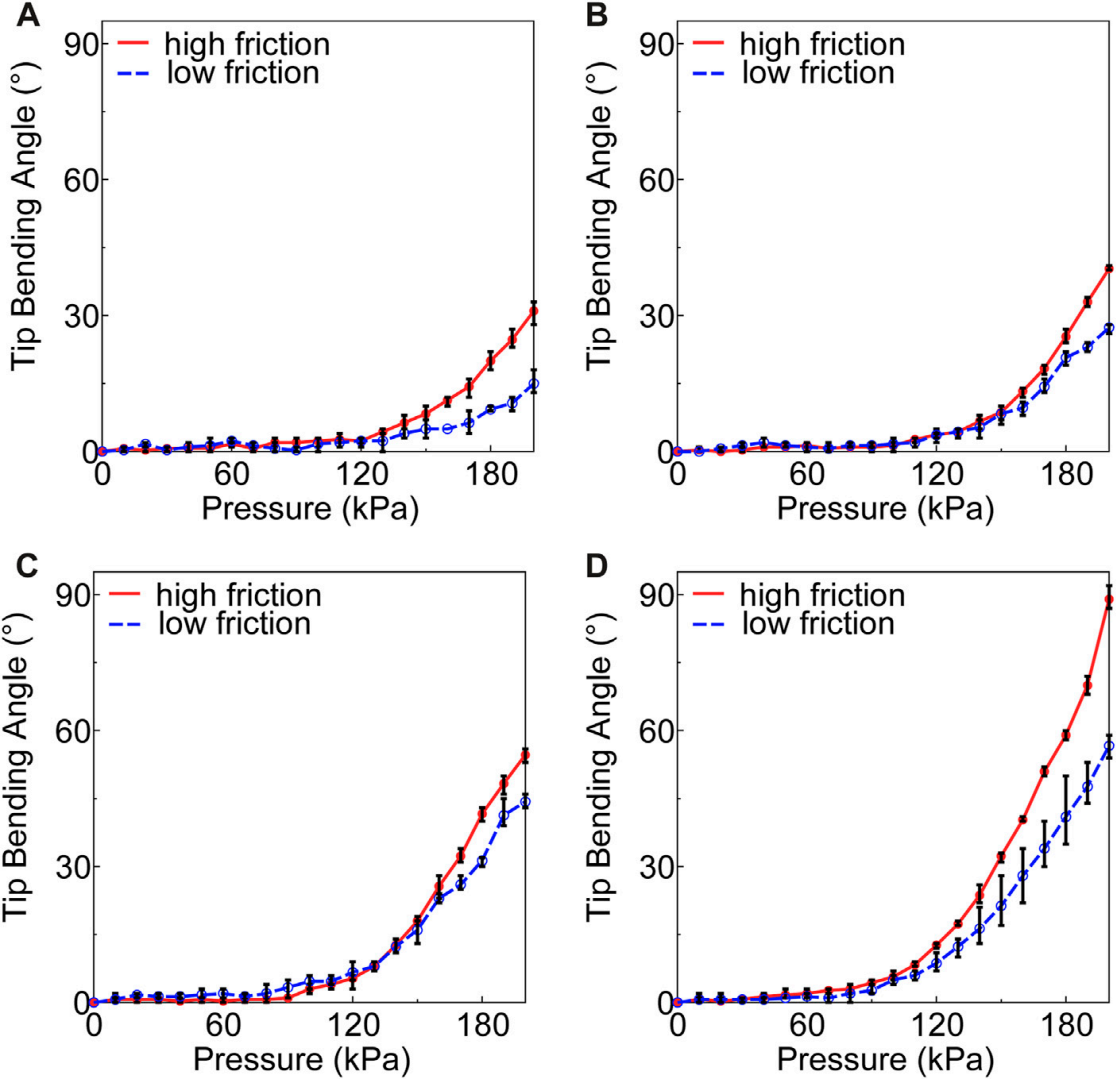
Air pressure variation of tip bending angle under 200 kPa when inserting endoscope on high friction and low friction with **(A)** circular, **(B)** fake crescent, **(C)** long fake crescent, **(D)** crescent.

Figure 5 shows the variation in the tip bending angle of the endoscope when inserted into soft actuators with circular, rectangular, fake crescent, long fake crescent, and crescent chamber shapes at 200 kPa in prototype experiments conducted under high- or low-friction conditions. The crescent-shaped chamber exhibited the largest tip-bending angle of the endoscope (crescent > long fake crescent > fake crescent > circular), similar to that of free bending.

In our hypothesis, we propose that low-friction conditions would enhance the actuator deformation and bending properties owing to the decreased resistance from the endoscope. In addition, we consider the potential of heightened friction to positively influence these properties. Our experimental data provide insights into this delineation. As shown in Figure 5, all the soft actuators, regardless of their chamber shapes, exhibited a more pronounced tip-bending angle under high-friction conditions than that under low-friction conditions. This suggests the pivotal role played by the increased friction force between the outer wall of the endoscope and the inner wall of the tube.

**FIGURE 6:**
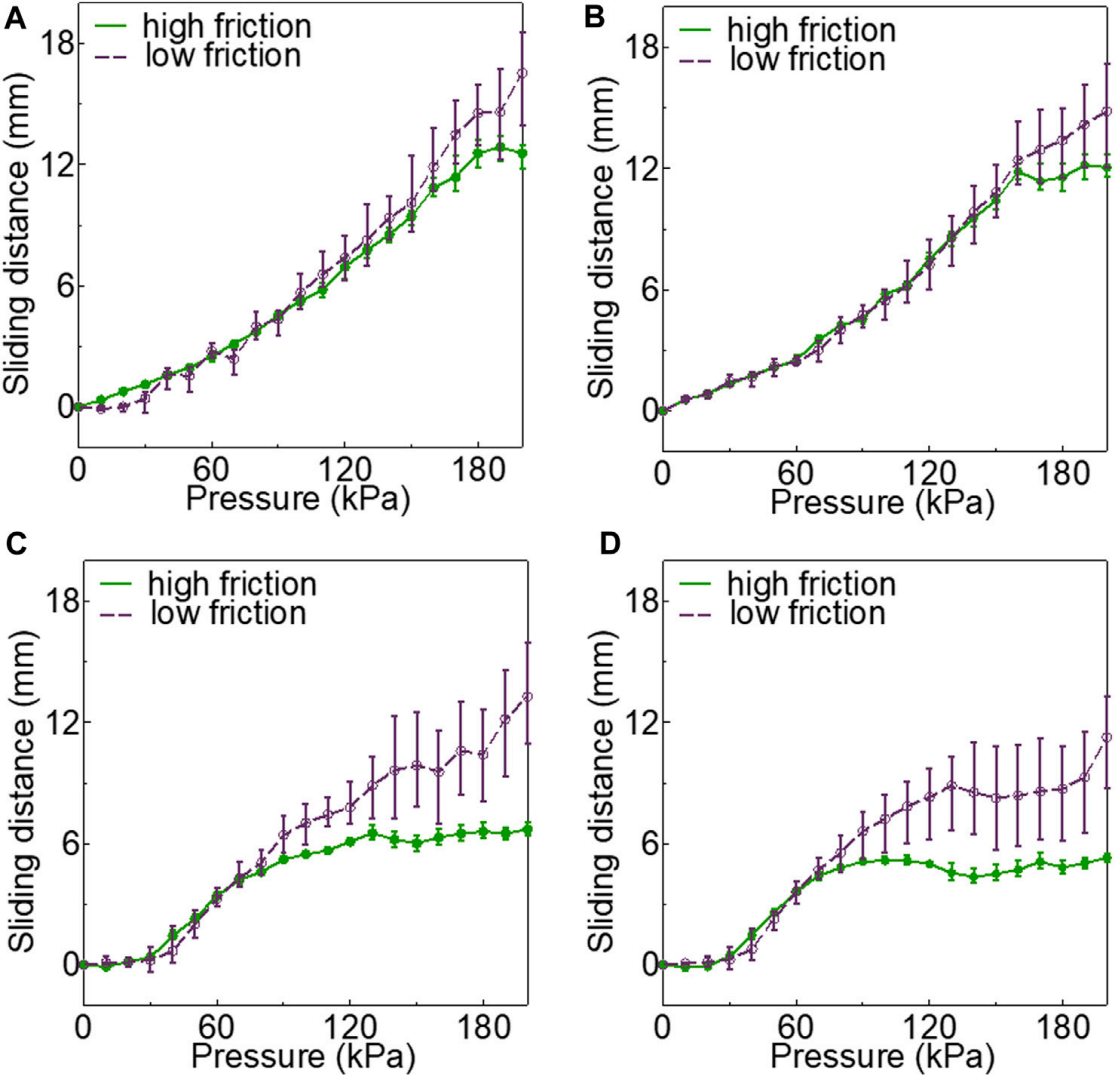
Air pressure variation of sliding distance under 200 kPa when inserting endoscope on high friction and low friction with **(A)** circular, **(B)** fake crescent, **(C)** long fake crescent, **(D)** crescent.

Furthermore, Figure 6 shows that the crescent-shaped chamber consistently had the shortest sliding distance under both friction conditions. This suggests that maximizing the friction can minimize sliding, subsequently affecting the bending properties of the actuator. These findings aligned with the initial considerations outlined in the hypotheses.

**FIGURE 7:**
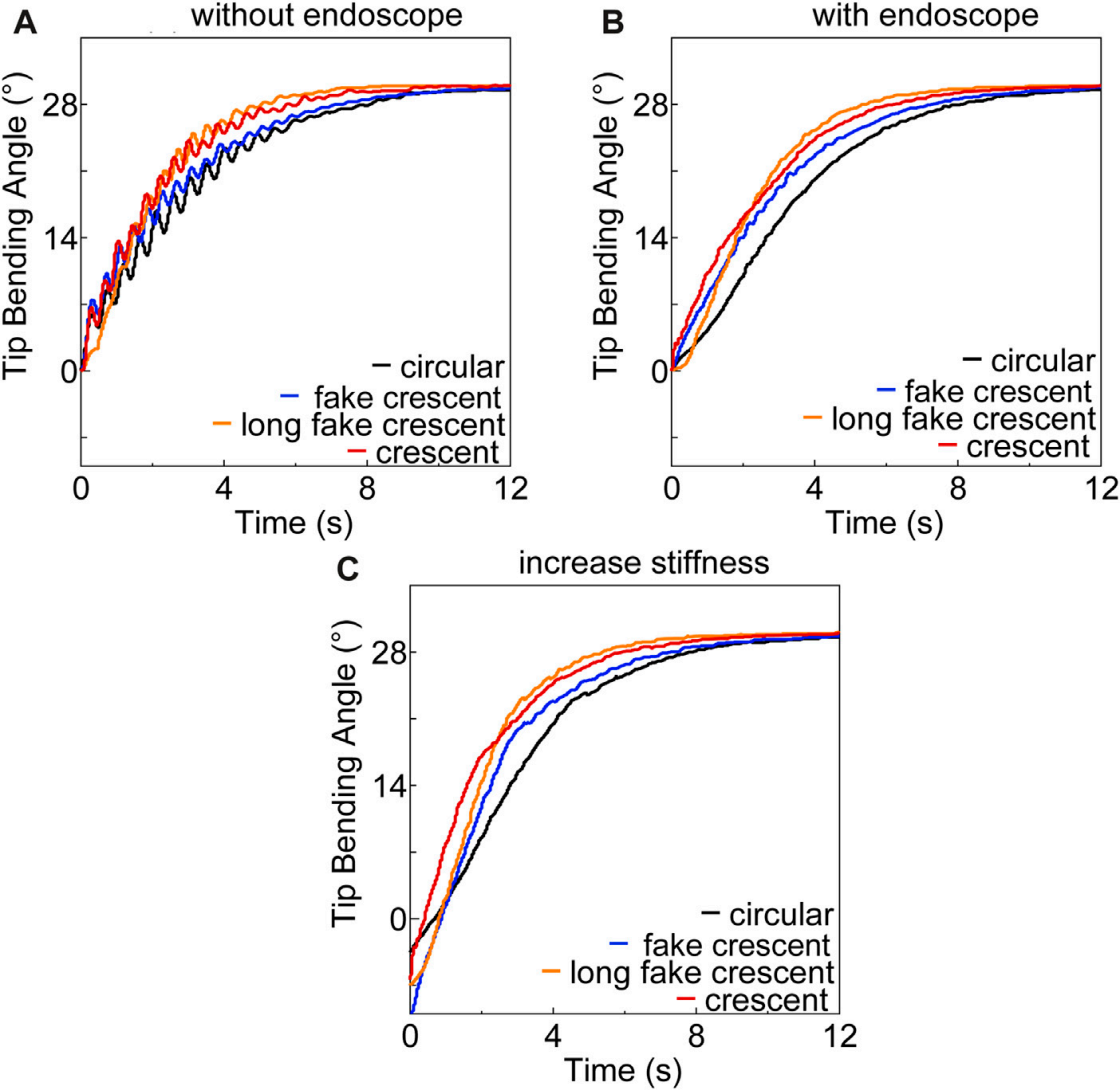
Performance of soft actuators with different pneumatic chamber **(A)** without endoscope **(B)** with endoscope **(C)** increase stiffness.

Figures 7A, B illustrate the step responses of soft actuators with different cross-sectional chamber shapes (circular, rectangular, fake crescent, long fake crescent, and crescent) using PID controllers for the free bending and endoscope insertion conditions, respectively. Table 2 presents the performance measures and PID gains of the PID controller for each actuator. The long fake crescent and crescent chamber shapes, both with wider widths, demonstrated faster response times in metrics, such as rising and settling times. The crescent and long-fake crescent chambers produced similar response times, likely owing to their similar chamber widths.

**TABLE 2:** Performance characteristics and PID gains of soft actuators.

	Without endoscope			With endoscope		
	$T_{r}\left(s\right)$	$T_{d}\left(s\right)$	$T_{a}\left(s\right)$	$T_{r}\left(s\right)$	$T_{d}\left(s\right)$	$T_{a}\left(s\right)$
Circular	6.111	2.061	8.443	6.382	2.828	8.773
Fake crescent	6.042	1.568	7.819	6.182	2.108	7.903
Long fake Crescent	3.882	1.540	5.374	4.021	1.954	5.815
Crescent	4.508	1.329	5.808	5.029	1.781	6.246

$T_{r}\left(s\right)$ , rising time; $T_{d}\left(s\right)$ , delay time; $T_{a}\left(s\right)$ , settling time.

Our results validated our hypothesis, underscoring the intricate relationship between chamber shape and actuator performance. Specifically, the influence of the chamber shape on the control performance of the actuator closely paralleled its effects on the bending properties. This interrelation is further exemplified by the observed deformation patterns and internal stress distribution, as illustrated in Figure 4E.

**FIGURE 8:**
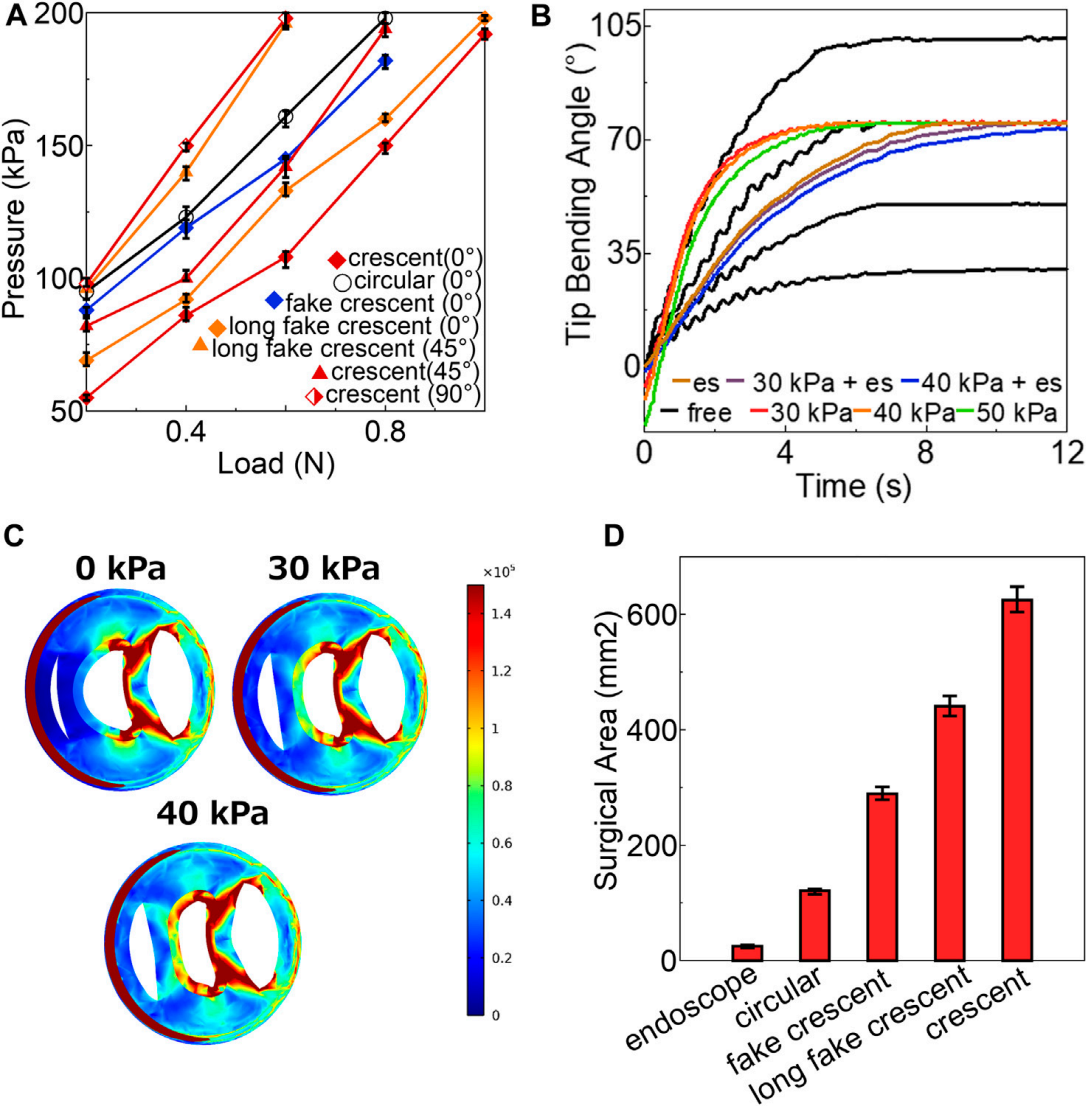
**(A)** The variation of stiffness adjustability under 1 N **(B)** Performance of soft actuators with crescent chamber **(C)** The stress distribution of cross-section of soft actuator with crescent chamber at 80 kPa **(D)** The surgical area for all soft actuators at 200 kPa.

The variation in the stiffness adjustability under a load capacity of 1 N is shown in Figure 8A. With the desired angle set to 0°, only actuators with crescent and long-fake crescent pneumatic chamber shapes could provide a 1 N load capacity. As the desired angle increased, the load capacity of all actuators decreased. Among them, the crescent-shaped actuator showed superior stiffness adjustability, capable of bending to all desired angles (0°, 45°, and 90°) under 0.6 N. Further insights from Supplementary Figure S3 reveal that heightened friction conditions bolster the stiffness adjustability across actuators of various chamber shapes: circular, rectangular, fake crescent, long fake crescent, and crescent. This finding underscores the significant role of friction in dictating actuator performance.

To address the unstable shaking issue observed in soft actuators, we inflated the opposite chamber to 30 kPa, which effectively enhanced the stiffness, as shown in Figure 7C. However, even when the opposite chamber of all actuators was maintained at < 30 kPa, the prototype performance remained suboptimal with persistent unstable shaking. The results of further tests on the maximum bending angle corresponding to the PID controller parameters outlined in Table 2 are presented in Figure 8B for free bending. Only the crescent-shaped chamber operated effectively at 50, 75, and 100 kPa using the specified PID controller settings. By contrast, the other actuators failed to reach the target bending angle without experiencing an overshoot, steady-state errors, or extended settling times beyond 10 s.

During the testing of the actuator with a crescent chamber shape, we found that increasing the air pressure in the opposite chamber above 30 kPa resulted in a reduction in the maximum tip bending angle to 75°. This observation led us to choose 75° as the desired angle for subsequent experiments, the results of which are depicted in Figure 8B. To mitigate the observed unstable shaking, we increased the stiffness of the soft actuators by pressurizing the opposite chamber (30, 40, and 50 kPa). Although this approach stabilized the actuator, the rising, delay, and settling times were extended by approximately 40%, compared with testing without the endoscope. However, any pressure >50 kPa in the opposite chamber rendered the soft actuator uncontrollable using the same PID controller parameters.

For all the tested soft actuators (circular, fake crescent, long fake crescent, and crescent), pressurizing the opposite chamber below 30 kPa did not resolve the issue of unstable shaking. However, inserting the endoscope improved the stability, which was attributable to increased stiffness. This insertion also resulted in an increase in the rising, delay, and settling times of the actuator. When air pressure was applied to the opposite chamber during endoscope insertion, the timings further escalated. Applying a pressure of more than 40 kPa in the opposite chamber prevented the soft actuator from reaching the target angle of 75°. Figure 8C shows the stress distribution of the crescent-shaped actuator at 80 kPa under varying pressures in the opposite chamber. We deduced that the increased pressure in the opposite chamber led to a reduced tube width, facilitating earlier contact between the tube and endoscope, thus restricting the expansion of the chamber. Despite these changes, the low-stress area remained largely consistent with the free-bending conditions (Figure 4).

Figure 8D shows the surgical areas of the different soft actuators, with the crescent-shaped chamber displaying the most expansive surgical area, which was six folds greater than that of the endoscope alone. This suggests that such an actuator can significantly improve the maneuverability of an endoscope, thereby yielding a broader surgical field. The data further underscore the significant influence of the chamber shape on the performance metrics and practical utility. Collectively, these results deepen our understanding of the impact of the pneumatic chamber design on the performance of soft actuators.

### 3.4 Prototype experiment for the soft actuator in a bladder model with flexible endoscope

In the bladder model experiment, we inserted a flexible endoscope with and without the crescent-shaped chamber soft actuator for comparison. The results are shown in Supplementary Video S1 (without an actuator) and Supplementary Video S2 (with an actuator). Without the actuator, controlling the flexible endoscope proved challenging, offering a limited view of the target surgical area (only 5 mm × 5 mm, with “L, A” visible). In contrast, when paired with the crescent-shaped chamber actuator, the bronchoscope demonstrated over 90° of bend and an “S” shaped two-degree freedom in conjunction with the inherent bronchoscope bending. This actuator facilitated rapid movement over the prostate (0.2 min by an inexperienced person in 10 trials) and offered a stable, expansive view of the target surgical area (25 mm × 25 mm, showing “L, A, R”). This configuration significantly augments the maneuverability and viewing radius of the endoscope. The crescent-shaped chamber actuator outperformed the surgical area by providing a field six folds larger than that of an endoscope alone.

## 4 Discussion

In our study on integrating endoscopes with soft actuators, we made an important observation: friction is crucial in the interaction between the soft actuator and the endoscope, which significantly affects the performance of the actuator. Actuators with wider chamber widths, such as crescent and long-fake crescent designs, exhibit increased friction, which in turn enhances actuator performance.

### 4.1 Bending property

With regard to the bending property, the FEA simulations revealed that the crescent-shaped chamber exhibited the most significant tip bending angle (Figure 4A). When the target angle was set to 15°, the circular chamber required an air pressure of approximately 80 kPa, whereas the crescent-shaped chamber required only 40 kPa. This suggests a potential 50% reduction in the air pressure required for the circular chamber, thereby enhancing safety. The primary driver behind the increased tip bending angle (Rus and Tolley, 2015) was the increase of chamber volume ( ∆V ), predominantly influenced by the chamber cross-sectional area increase (Figure 4C).

Additionally, the change in chamber height was significantly greater than that in chamber width (as shown in Figure 4C). This was attributed to the fact that the hollow part was more prone to compression, causing the chamber to expand towards the hollow part of the soft actuator (tube) rather than outward and sideways when the outer part of the soft actuator was strongly bonded by the nylon fiber. A wider chamber width resulted in a low-stress area in the direction of the hollow part of the soft actuator (as depicted in Figure 4E), allowing the chamber to expand easily towards the hollow part (tube) and increase the chamber height while obtaining a larger chamber cross-sectional area. The crescent-shaped chamber, which had the widest chamber width, exhibited the largest low-stress area and tip bending angle. Moreover, the crescent chamber showed the lowest stress in A, B, C, and D under a low tip-bending angle (Supplementary Figure S1). This suggests that the crescent chamber structure is more conducive to rapid and large deformations by air pressure than the other chambers. By contrast, the soft actuator with a circular chamber exhibited the largest stress among the soft actuators in A, B, C, and D at the same tip-bending angle. This indicates that it is more challenging for the circular chamber to rapidly and substantially deform under air pressure.

As observed, the endoscope underwent relative sliding with the soft actuator during bending. Given the inherent stiffness of the endoscope, which surpasses that of the soft actuator, it resists the bending motion of the soft actuator. This behavior implies that the input air pressure in the inflated pneumatic chamber was largely directed towards elongating the chamber in the direction of the endoscope, culminating in relative sliding rather than genuine bending.

Delving deeper into the design intricacies, a wider chamber width, as exemplified by the crescent-shaped soft actuator, facilitated a more pronounced expansion of the pneumatic chamber in the actuator’s hollow region. This design feature results in a greater contact pressure on the endoscope, thus intensifying the friction between the outer wall of the endoscope and the inner wall of the tube. Our friction tests, shown in Figure 7, further confirm this observation, highlighting that increased friction can significantly reduce the sliding distance between the endoscope and soft actuator. In essence, such heightened friction restricts the elongation of the inflatable chamber towards the endoscope, fostering greater expansion towards the hollow section, and thereby yielding a sharper tip bending angle of the endoscope.

However, the design efficiency has inherent challenges. Despite their efficiency, crescent-shaped actuators tend to compress the tube (Figures 4D, E), posing potential challenges during surgical procedures that require frequent instrument changes. Although lubricants or low-friction tubes might ease this challenge, they could marginally compromise the bending efficiency. Circular-chambered actuators, although perhaps inferior in bending capability, may offer a balanced solution owing to their structural attributes.

Our research investigates the influence of cross-sectional shapes of pneumatic chambers in the bending property of soft actuators, a critical element in enhancing the functionality of tools used in MIS. The findings demonstrate that crescent-shaped chambers exhibit improved bending properties and require lower operational air pressure, thereby increasing safety and efficiency in surgical settings. This observation aligns with the principles outlined (Rus and Tolley, 2015), underscoring the importance of actuator design in medical applications. Additionally, our study sheds light on the role of friction in actuator performance, emphasizing the need to control the interaction between the actuator and the endoscope. This aspect of our research offers insights into material and design considerations for soft actuators, contributing to the broader field of soft robotics and surgical technology as discussed in the works (Heung et al., 2020; Lee et al., 2017). By focusing on these specific design elements, our study adds to the understanding of how soft actuators can be optimized for MIS, thus advancing the development of more effective medical robotic tools.

### 4.2 Stiffness adjustability

Second, in exploring the stiffness adjustability, we found that bilateral symmetrical air chambers are essential in soft actuator design. Bilateral symmetrical air chambers are indispensable for optimizing soft actuator design. Notably, the actuator with the crescent cross-sectional chamber exhibited remarkable stiffness adjustability (Figure 8A). This enhanced stiffness bolsters the resilience of the actuator against external pressures, while also refining the control performance of the PID controller, as shown in Figures 7, 8B. This capability underscores the potential of these soft actuators in maintaining a steady posture when deployed within the human body, thereby demonstrating their invaluable utility in various surgical procedures.

Based on our findings, as detailed in Supplementary Figure S3, it is evident that friction plays a central role in the performance of soft actuators. Specifically, under high-friction conditions, soft actuators of all cross-sectional chamber shapes—circular, rectangular, fake crescent, long fake crescent, and crescent—demonstrated an enhanced stiffness adjustability. Given the significance of these observations, future research should focus on effectively integrating frictional behavior into actuator design. This could entail refining the structural design of actuators, exploring materials with specific frictional properties, and devising control strategies that leverage frictional dynamics.

Although these findings highlight the potential of soft actuators, they introduce certain constraints. However, a notable limitation emerged when the air pressure in the opposing chamber exceeded 30 kPa. Under such conditions, the tip-bending angle of the soft actuator was reduced by over 25%, compared with its performance during free bending. Additionally, there is a risk of chamber rupture, and this could be attributed to the opposing side of the air chamber encroaching on the internal tube spaces. During bending, the immense internal pressure causes the air chambers on either side to exert forces against each other, as shown in Figure 8C. Such insights are crucial for ensuring the safe and effective application of soft actuators in surgical environments.

In our study on soft actuators for MIS, we have identified stiffness adjustability as a key factor influencing their performance and safety, a notion resonating with current trends in soft robotics and surgical technology. The interaction dynamics between soft actuators and endoscopes, particularly in terms of material stiffness and friction, are central to this finding. Echoing the research (Runciman et al., 2019; Xavier et al., 2021), we highlight the critical role of material properties in these interactions. Furthermore, our introduction of bilateral symmetrical air chambers, supported by previous studies (Stilli et al., 2014; Lu et al., 2023), represents a significant advancement in enhancing stiffness adjustability. This design not only bolsters resilience against external pressures but also optimizes control performance, crucial for the efficacy and safety of soft actuators in surgical applications. Overall, our findings contribute to a deeper understanding of soft actuator design in MIS, underscoring the importance of integrating material and structural considerations for improved surgical outcomes.

### 4.3 Control property

As earlier discussed, actuators with wider chambers, such as crescent and long-fake crescent designs, have significantly improved bending property and frictional force. This improvement directly leads to faster response times, which are crucial in surgical applications.

With regard to the response properties, the empirical data from our experiments (Table 2) reveal that a soft actuator with a crescent cross-sectional chamber shape is superior in terms of the delay time and overall bending properties. Although the rising and settling times were marginally surpassed by that of the long-fake crescent design, its holistic performance was remarkable. The stability of a crescent actuator is a defining trait, as illustrated in Figure 8B. The pronounced friction in this design ensures enduring and stable endoscope-tube interactions, substantially reducing overshoot risks.

At the other end of the spectrum, soft actuators with lower friction profiles, such as those with circular and fake crescent cross sections present certain challenges. Their predisposition to increased sliding distances culminates in an unstable interaction between the tube and endoscope. This instability not only leads to overshoot but also complicates the calibration of the PID controller to its optimal parameters. Furthermore, the bending capabilities of these low-friction actuators are somewhat constrained and often fall short when tasked with achieving more pronounced desired angles.

Our research elucidates the intricate interplay between chamber design, stiffness adjustability, and control properties of soft actuators. The crescent-chambered actuator offers improved stiffness and control, particularly when modulating the air pressure in the opposing chamber. Based on our data, as shown in Figure 8B and detailed in Supplementary Table S2, there is a critical threshold for maintaining the air pressure of the opposing chamber between 30 and 50 kPa. Beyond this, the efficacy of the PID controller is diminished.

For endoscopes with a radius exceeding 3.9/2 mm, increasing the air pressure above 30 kPa in the opposite chamber is not recommended. The underlying concern is the risk of premature tube-endoscope contact triggered by heightened pressure. Such interactions can restrict the expansion of the inflated chamber, compromising both the bending range of the tip and the control capabilities of the PID controller.

Our study’s exploration of soft actuators in MIS reveals crucial insights into the role of chamber design and frictional dynamics in enhancing response properties. We discovered that actuators with wider chambers, such as the crescent design, exhibit markedly improved bending properties and frictional forces, leading to faster and more reliable response times. This improvement is particularly significant in the precise and demanding environment of MIS, where the speed and accuracy of actuator response directly impact surgical outcomes. These findings not only contribute to the evolving field of soft robotics in surgical technology but also align with the broader pursuit of more effective and responsive surgical aids. The interplay between chamber design, frictional forces, and response characteristics underscores the importance of considering these factors in the development of next-generation soft robotic systems for MIS, reaffirming our study’s relevance and contribution to the field.

From our findings, it is evident that design intricacies and functional outcomes are intertwined. The chamber shape and frictional dynamics are paramount, emphasizing their role in optimizing soft actuators for MIS applications.

Finally, we observed that the soft actuator notably enhanced the maneuverability and bending radius of the endoscope, as evidenced in Figure 8D; Supplementary Videos S1, S2. This improved freedom empowers the endoscope to navigate around obstructions with ease, mitigating potential organ damage due to collisions and ensuring a stable surgical view. These capabilities underscore the potential of soft actuators in facilitating more intricate MIS procedures.

### 4.4 Contributions

1. Elucidation of Chamber Shapes: Our study elucidated the role of cross-sectional shapes in pneumatic chambers, revealing their influence on the bending properties, response dynamics, and stiffness adjustability of soft actuators.
2. Emphasis on Frictional Forces: We highlight the pivotal role of frictional forces stemming from chamber interactions as a key determinant of actuator performance.
3. Design vs. Performance: Our findings underscore the delicate balance between actuator design nuances and the resultant operational performance.
4. Advancements in MIS: In the field of MIS, our research highlights avenues for heightened surgical precision. This emphasizes the need for designs that augment patient safety and procedural efficiency.
5. Reference for future designs: This study serves as a crucial reference for guiding future innovations in soft actuator design and applications as MIS technology evolves.

### 4.5 Limitations

1. Inter-chamber interference: The potential interference between multiple antagonistic air-chamber pairs has not been fully explored.
2. Endoscope dimensions and shape: Our study did not extensively investigate the impact of varying the external diameters and shapes of the inserted endoscopes on the actuator performance.
3. Frictional consistency: While friction has been identified as a key factor, the consistency of friction over prolonged use and under varied conditions remains uncharted.

## 5 Conclusion

Although the significance of chamber design in soft actuators for MIS has always been recognized, our findings highlight an unexpected advantage. We found that a wider chamber width not only influences the interaction with endoscopes, but also amplifies the pressing and resultant friction forces. This enhancement directly translates into an expanded range of motion and precision for navigating endoscopic instruments.

Among the evaluated designs, the soft actuator with a crescent-shaped chamber was notably superior. Characterized by its extensive chamber width and the inclusion of a bilateral symmetrical air chamber, this configuration exhibits exceptional bending and response properties without compromising patient safety. The intrinsic adjustable stiffness of this design offers versatility, making it well-suited for a diverse range of surgical applications.

These insights provide a deeper understanding of the factors crucial for the design of endoscope-supporting soft actuators and offer directions for future research. Our next step involved animal testing to further assess the practical capabilities of endoscopes paired with crescent-shaped soft actuators. We hope that our ongoing research will shed more light on the potential benefits of soft actuators for enhancing MIS procedures.

## Data Availability

The original contributions presented in the study are included in the article/Supplementary Material, further inquiries can be directed to the corresponding author.
