# Supplementary material for: Effects of chamber shapes on maneuverability and control property of endoscope-support soft actuators
Source: Front Bioeng Biotechnol. 2023 Dec 14;11:1319922. doi: 10.3389/fbioe.2023.1319922 (PMC10757984; doi:10.3389/fbioe.2023.1319922)
Supplement: Supplementary file 3 [file DataSheet1.docx]

Supplementary Material

# Design and for Soft Actuators

**Table S1** The parameters of soft actuator.

| chamber name | S (mm^2^) | $W_{c}$ (mm) | $W_{t}$ (mm) | $H_{c}$ (mm) | L (mm) |
| --- | --- | --- | --- | --- | --- |
| circular | 6.282 (2π) | 2.828 | 6.000 | 2.828 | 100.000 |
| semicircular | 6.282 (2π) | 4.000 | 6.000 | 2.000 | 100.000 |
| square | 6.282 (2π) | 2.507 | 6.000 | 2.507 | 100.000 |
| rectangular | 6.282 (2π) | 6.283 | 6.000 | 1.000 | 100.000 |
| fake crescent | 6.282 (2π) | 3.107 | 6.000 | 2.000 | 100.000 |
| long fake crescent | 6.282 (2π) | 6.022 | 6.000 | 1.000 | 100.000 |
| crescent | 6.282 (2π) | 9.037 | 6.000 | 1.000 | 100.000 |

# Fabrication of the Soft Actuator

The soft actuators were fabricated using molds printed with a 3D-printer (Ultimaker 2 Extended+, Ultimaker B.V., Netherlands) using PLA filament. Dragonskin 10 MEDIUM (Smooth-On, Inc., US) was used to fabricate the main bodies of the soft actuators. Dragonskin 10 MEDIUM consisted of two parts mixed together at a 50:50 mass ratio. The mixture was subjected to vacuum for 15 min to de-air and then slowly poured into appropriate molds. Dragonskin 10 MEDIUM was used as the final coating to fix the strain-limiting fibers (Kevlar thread; major radius:8.65 mm, minor radius,0.483 mm; axial pitch,2 mm; number of turns,59; CS Hyde Co., USA) with helix restraint. A silicon tube (major radius:4 mm; minor radius:3 mm; Dragonskin 30, Smooth-On, Inc., US) was placed in the middle of the soft actuators.

# FEA Simulations


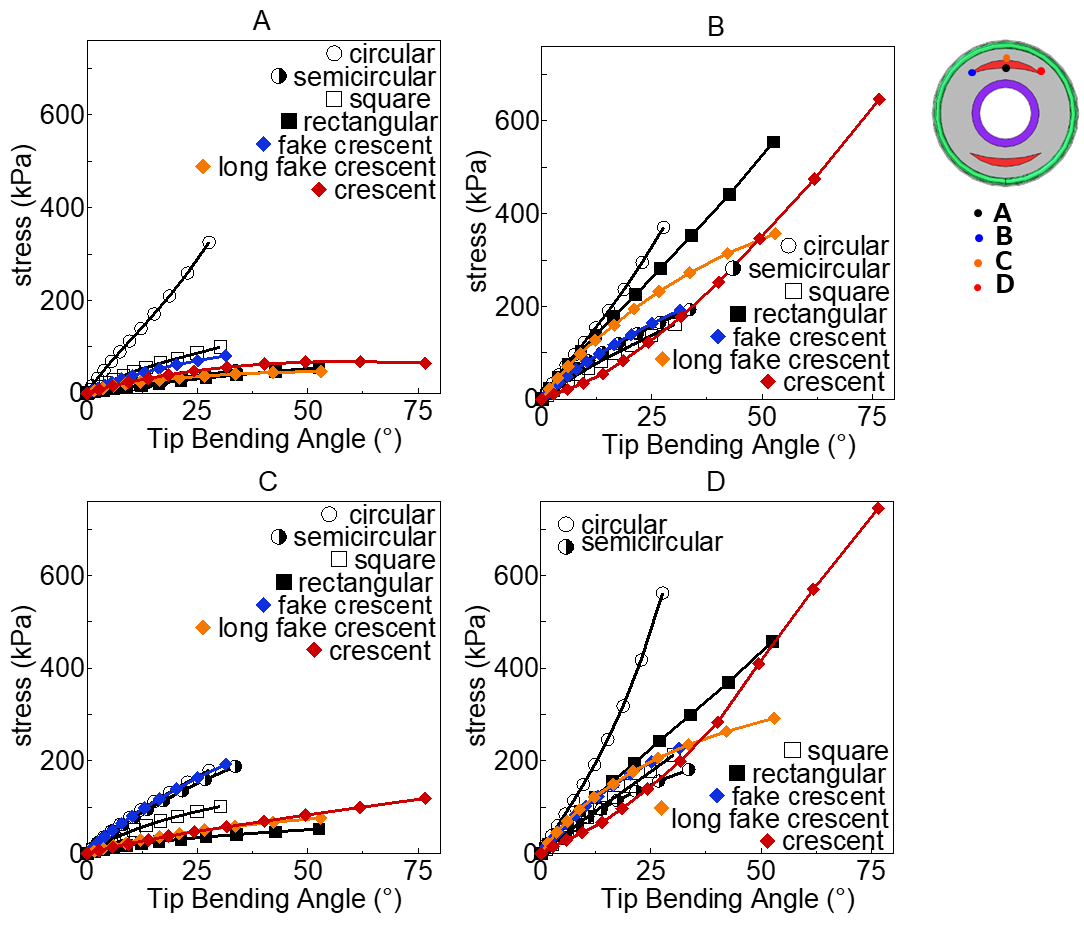


**Figure S1** The variation of stress in A, B, C, D under different tip bending

# Prototype experiments for free bending

**Figure S2** Air pressure variation of tip bending angle under 200 kPa on FEM simulations and Prototype experiments test for free bending angle (a)circular, (b)fake crescent, (c) long fake crescent, (d) crescent

# Prototype Experiments for Bending Property, Control Performance and Stiffness Adjustability of the Soft Actuator

**Figure S3** The variation of stiffness adjustability under at 0° with 1 N (a)circular, (b)fake crescent, (c) long fake crescent, (d) crescent

**Table S2.** The performance characteristics and PID gains of the soft actuator with a crescent chamber

| name | air pressure in the opposite chamber (kPa) | $T_{r}(s)$ | $T_{d}(s)$ | $T_{a}(s)$ |
| --- | --- | --- | --- | --- |
| Antag_0 | 0 | 4.497 | 1.967 | 5.200 |
| Antag_30 | 30 | 2.513 | 0.960 | 3.514 |
| Antag_40 | 40 | 2.634 | 1.000 | 3.594 |
| Antag_50 | 50 | 3.074 | 1.040 | 4.358 |
| endoscope (es) | 0 | 5.640 | 2.400 | 6.810 |
| Antag_30 + es | 30 | 6.240 | 2.490 | 8.010 |
| Antag_40 + es | 40 | 7.560 | 2.640 | 9.660 |

$T_{r}(s)$: rising time, $T_{d}(s)$: delay time, $T_{a}(s)$: settling time.
